# A170 ADVERSE EVENTS & SEROLOGICAL RESPONSES FOLLOWING SARS-COV-2 VACCINATION IN INDIVIDUALS WITH INFLAMMATORY BOWEL DISEASE

**DOI:** 10.1093/jcag/gwac036.170

**Published:** 2023-03-07

**Authors:** A Markovinovic, M Herauf, J Quan, L Hracs, J W Windsor, N Sharifi, S Coward, L Caplan, J Gorospe, C Ma, R Panaccione, R Ingram, J Kanji, G Tipples, J Holodinsky, C Berstein, D Mahoney, S Bernatsky, E Benchimol, G G Kaplan

**Affiliations:** 1 University of Calgary, Calgary; 2 McGill University, Montreal; 3 University of Toronto, Toronto, Canada

## Abstract

**Background:**

The rapid development and distribution of SARS-CoV-2 vaccines has raised concerns surrounding vaccine safety in immunocompromised populations, such as those with inflammatory bowel disease (IBD).

**Purpose:**

We described adverse events (AEs) following SARS-CoV-2 vaccination in those with IBD and determined relationships between AEs to post-vaccination antibody titres.

**Method:**

Individuals with IBD from a prospective cohort in Calgary, Canada (*n*=670) who received a 1^st^, 2^nd^, 3^rd^, and/or 4^th^ dose of a SARS-CoV-2 vaccine (Pfizer-BioNTech, Moderna, and/or AstraZeneca) were interviewed via telephone for AEs using the Adverse Events Following Immunization form. Subsequently, we assessed injection site reaction as a specific AE outcome. Multivariable logistic regression models were used to assess the association between anti-SARS-CoV-2 spike protein antibody (anti-S) levels within 1–12 weeks of vaccination and injection site reaction following 1st, 2nd, and 3rd dose vaccination. Models were adjusted for age, sex, IBD type, IBD medications, vaccine type, and prior COVID-19 infection. Additionally, we evaluated the risk of flare of IBD within 30 days of vaccination via chart review.

**Result(s):**

Table 1 describes AEs in individuals with IBD following 1^st^ dose (*n*=331), 2^nd^ dose (*n*=331), 3^rd^ dose (*n*=195), and 4^th^ dose (*n*=100) of a SARS-CoV-2 vaccine. AEs were reported in 83.3% of participants after 1^st^ dose, 79.1% after 2^nd^ dose, 77.4% after 3^rd^ dose, and 67.0% after 4^th^ dose. Injection site reaction (pain, redness, etc.) was the most common AE (50.8% of AEs), with fatigue and malaise (18.1%), headache and migraine (8.6%), musculoskeletal discomfort (8.2%), and fever and chills (6.5%) also commonly reported. Multivariable logistic regression determined no associations between anti-S concentration and injection site reaction for all doses. Age above 65 years was associated with decreased injection site reaction following 1^st^ and 3^rd^ doses, while female sex and mRNA vaccine type were associated with increased injection site reaction following 1^st^ and 2^nd^ doses. Prior COVID-19 infection, IBD type, and medication class were not associated with injection site reaction with any dose. Only one participant was diagnosed with a severe AE requiring hospitalization: Immune thrombocytopenic purpura (ITP) following 2^nd^ dose of a Pfizer vaccination. No cases of IBD flare occurred within 30 days of vaccination.

**Image:**

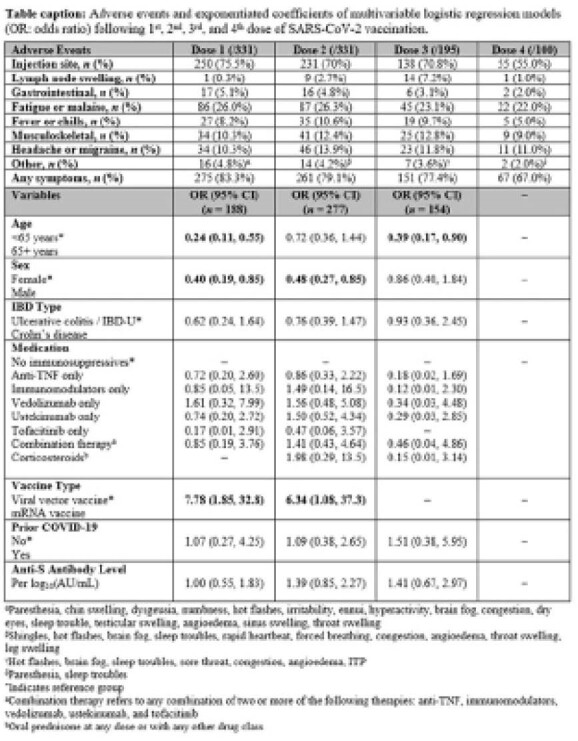

**Conclusion(s):**

AEs following SARS-CoV-2 vaccination are generally mild and become less common with each consecutive dose. Antibody levels following each dose of the vaccine were not associated with injection site reactions. Females, those under 65 years of age, and those administered mRNA vaccines were more likely to experience an injection site reaction. Prior COVID-19 infection, IBD type, and IBD medication class did not predict injection site reactions. Vaccination was not associated with IBD flare within 30 days of vaccination.

**Please acknowledge all funding agencies by checking the applicable boxes below:**

Other

**Please indicate your source of funding;:**

Helmsley

**Disclosure of Interest:**

A. Markovinovic: None Declared, M. Herauf: None Declared, J. Quan: None Declared, L. Hracs: None Declared, J. Windsor: None Declared, N. Sharifi: None Declared, S. Coward: None Declared, L. Caplan: None Declared, J. Gorospe: None Declared, C. Ma Grant / Research support from: Ferring, Pfizer, , Consultant of: AbbVie, Alimentiv, Amgen, Ferring, Pfizer, Takeda, , Speakers bureau of: AbbVie, Alimentiv, Amgen, Ferring, Pfizer, Takeda, R. Panaccione Grant / Research support from: AbbVie, Ferring, Janssen, Pfizer, Takeda, Consultant of: Abbott, AbbVie, Alimentiv, Amgen, Arena, AstraZeneca, Boehringer Ingelheim, Bristol Myers Squibb, Celgene, Celltrion, Cosmos Pharmaceuticals, Eisai, Elan, Eli Lilly, Ferring, Galapagos, Genentech, Gilead Sciences, GlaxoSmithKline, Janssen, Merck, Mylan, Oppilan Pharma, Pandion Therapeutics, Pandion Pharma, Pfizer, Progenity, Protagonist, Roche, Sandoz, Satisfai Health, Schering-Plough, Shire, Sublimity Therapeutics, Takeda, Theravance, UCB, Speakers bureau of: AbbVie, Arena, Celgene, Eli Lilly, Ferring, Gilead Sciences, Janssen, Merck, Pfizer, Roche, Sandoz, Shire, Takeda, R. Ingram: None Declared, J. Kanji: None Declared, G. Tipples: None Declared, J. Holodinsky: None Declared, C. Berstein Grant / Research support from: AbbVie, Amgen, Janssen, Pfizer, Takeda, Speakers bureau of: AbbVie, Janssen, Pfizer, Takeda, D. Mahoney: None Declared, S. Bernatsky: None Declared, E. Benchimol: None Declared, G. Kaplan Grant / Research support from: Ferring, Speakers bureau of: AbbVie, Janssen, Pfizer

